# Turning Everything Upside Down: The Impact of Illness on Romantic Relationships—A SEM‐Based Actor‐Partner Interdependence Model

**DOI:** 10.1111/famp.70129

**Published:** 2026-02-13

**Authors:** Franziska Reinhardt, Imad Maatouk

**Affiliations:** ^1^ Department of Internal Medicine, Division of Integrated Psychosomatic Medicine University Hospital Wuerzburg Wuerzburg Germany

**Keywords:** actor‐partner interdependence model, chronic illness, dyadic coping, relationship satisfaction

## Abstract

Illnesses are often associated with fundamental changes in relationship dynamics. Yet, little is known about how different acute and chronic conditions distinctly impact both partners' well‐being. This study analyzed the reciprocal associations between different illnesses on life satisfaction and relationship satisfaction in partnerships. Based on data from the Family Demographic Panel (FReDA), 14,426 people were analyzed in a two‐stage analysis process. In the first step, a Welch‐ANOVA examined disease‐specific differences in life satisfaction as a function of partnership status, focusing on the well‐being of the ill individual. In the second step, an extended Actor–Partner Interdependence Model (APIM) was applied to partnered dyads, shifting the focus to the healthy partner and analyzing how the illness of one partner and relational factors are associated with the partner's relationship and life satisfaction. The results revealed substantial crossover effects, with consistent partnership‐related patterns across illness types. Disease severity and duration were negatively associated with relationship satisfaction, whereas intimacy and constructive conflict resolution emerged as universal protective factors. These findings underscore the relevance of a systemic perspective in healthcare and show the need for partnership‐oriented interventions.

## 1 Introduction

Imagine you are living in a happy partnership when you are suddenly diagnosed with an illness. This situation can change everything: routines, priorities and even the dynamics of your relationship. Studies show that health challenges are associated with physical, emotional, psychosocial and economic stress for both partners (Jaspers et al. 2015; Rohrbaugh 2021).

### 1.1 Partnership Function and Health Mechanisms

The stabilizing function of partnerships for health is well documented: People in relationships show better recovery trajectories and higher survival rates for serious illnesses (Holt‐Lunstad et al. 2010). Although the partnership can provide a framework for coping together, a diagnosis also always means changes within the partnership, such as shifts in role dynamics and responsibilities (Pietromonaco et al. 2013).

These patterns highlight the importance of examining the complex interactions between illness and partnership more closely. A better understanding of these dynamics can lead to more effective support strategies for affected couples and thus improve the quality of life of both partners. Therefore, the present study investigated how different illnesses are associated with life satisfaction and relationship satisfaction in couples. By comparing several diagnostic groups, it sought to identify both general and illness‐specific patterns in the interplay between health and partnership.

Partnerships fulfill several central functions that contribute significantly to coping with illness. One important function is co‐regulation, in which partners jointly regulate their emotional states. This supports functional emotion regulation, which is crucial for psychological well‐being (Butler 2011). These synchronized physiological processes not only influence metabolic and neuroendocrine factors, but may even have epigenetic effects, as shown in animal models of social isolation (Arzate‐Mejía et al. 2020). Close relationship partners also promote the fulfillment of individual needs and thus contribute significantly to general well‐being (Holt‐Lunstad and Steptoe 2022). This positive association co‐occurs with healthy lifestyle habits such as sleep, diet, and exercise, which correlate with social support patterns (Proctor et al. 2023). Analyzing the relationship between partnership and health reveals that the positive function of partnerships goes far beyond immediate emotional support and has significant influences on physical health and well‐being.

Bodenmann's systemic transactional model provides a strong theoretical foundation for this. It describes that at low stress levels, individual coping mechanisms are often sufficient to regulate stress. However, as stress levels increase in the context of chronic illness or persistent external stressors, dyadic coping patterns emerge more prominently. In this context, a shared *we‐stress* experience emerges, which not only alleviates individual stress responses but also strengthens the bond between partners (see Bodenmann 1995; Bodenmann et al. 2016). Building on this dyadic perspective, Umberson et al. (2010) further highlight that partnerships are embedded in wider social and structural contexts that influence health behavior, stress exposure, and overall well‐being. Their conceptual model demonstrates how social ties shape health through multiple interrelated pathways, including social control, emotional support, stress processes, and physiological as well as psychological responses.

### 1.2 Specific Illnesses and Their Individual Impact on the Partnership

The type and characteristics of an illness have a significant impact on how couples have to deal with it (Berg and Upchurch 2007; Gallant 2003).

Cancer poses particularly complex challenges for couples. Stress experiences and emotional adaptation processes show strong associations in both partners (Northouse et al. 1995). The central mechanisms here are communication between partners and emotional attachment, which can influence the recovery process (Manne and Badr 2008). Even after treatment completion, fears of recurrence and long‐term psychological stress are frequently reported in the partners of patients (Hodgkinson et al. 2007). Cancer treatment also has lasting effects on relationship quality, particularly in relation to changes in sexuality and physical appearance (Di Mattei et al. 2021).

Cardiovascular diseases, especially heart attacks, usually occur suddenly and are often life‐threatening events that require immediate adaptation (Friedman and Quinn 2008). Following such a crisis, partners tend to engage in overprotective behavior, which is referred to as cardiac invalidity (Clarke et al. 1996). This overprotective behavior could affect a partnership by leading to role reversal or additional tasks for the partner (Dalteg et al. 2011). Studies also show that couples who cling to the status quo often have lower satisfaction levels than those who experience the disease as threatening but actively try to cope with it (Bertschi et al. 2021; Mahrer‐Imhof et al. 2007).

Chronic illnesses such as renal insufficiency or respiratory diseases, on the other hand, require long‐term adjustments to daily life. Couples facing chronic illness often report establishing new routines, redistributing roles, and integrating time‐consuming medical treatments (Meier et al. 2012). In addition to overprotective behavior, the phenomenon of *protective buffering* is often evident here; healthy partners try to protect their partner by hiding their own worries and avoiding illness‐related topics (Snippe et al. 2012). However, research shows that focusing on positive goals combined with active partner support reduces psychological stress better than an avoidance‐oriented strategy (Schokker et al. 2010). As the disease progresses, the role of the healthy partner also expands: as part of the safety network, they often develop a particular sensitivity to critical health conditions (Mackintosh et al. 2020). Over time, partners frequently develop expertise through continuous engagement with treatment and symptom management (Sormanti and Kayser 2000). Although these changed roles and responsibilities can place a burden on both partners (Pietromonaco et al. 2013), coping together also harbors opportunities: the established daily structures and routines not only contribute to better coping with the illness, but can also strengthen the partnership's sense of togetherness (Bertschi et al. 2021).

Musculoskeletal disorders and fractures pose particular practical challenges for couples. The acute phase of these illnesses often leads to an intense, albeit usually temporary, burden on the partners, who suddenly have to provide a high level of day‐to‐day support (Ariza‐Vega et al. 2019). This burden is further exacerbated by financial losses, particularly due to work impairment and decreased productivity (Soen et al. 2021). The resulting temporary or permanent dependencies require a redistribution of roles and responsibilities, which can permanently shift the established balance in the partnership (Parry et al. 2019).

Lifestyle‐associated diseases such as diabetes or high blood pressure have a special dynamic: partners can have a direct influence on the course of the disease through joint behavioral changes. Research shows that adherence to treatment plans and lifestyle changes depends largely on support from partners (Gallant 2003). Social control and mutual motivation play a particularly important role in establishing healthy routines in areas such as sleep, diet, and exercise (Proctor et al. 2023). At the same time, different health beliefs and behaviors between partners can lead to conflict (Glasgow et al. 2001).

Mental illnesses hold a special position, as they directly influence the relationship dynamics. Mental illnesses show distinct association patterns with fundamental relationship aspects compared to physical illnesses, such as emotional interaction, intimacy, and communication (Butler 2011). The interaction between relationship and illness is particularly complex: relationship factors are associated with both the development and maintenance of mental disorders (Whisman 2006). An additional burden arises from the social stigmatization of mental illness. This stigma not only affects those diagnosed but also extends to their relatives as *courtesy stigma*, exposing them to both direct discrimination and the marginalization of their partners, factors that significantly impact their own quality of life (Angermeyer et al. 2003).

Across different illness types, research shows that couples face diverse challenges but rely on similar relational processes. Communication, emotional closeness, and joint coping are key for how partners adapt and maintain satisfaction. The specific features of each illness such as its onset, duration, or visibility determine how strongly couples need to adjust their daily lives and relationship roles.

However, the extent of this impact likely depends on characteristics of the partnership itself. Relationship status, quality, and available partnership resources may moderate how couples experience and adapt to illness. Examining these moderating dynamics is therefore essential for understanding how illness and partnership interact across different health conditions.

These interrelations can be theoretically understood within the developmental–contextual model of couples coping with chronic illness proposed by Berg and Upchurch (2007). The model conceptualizes chronic illness as a shared developmental process in which individual, dyadic, and contextual factors continuously interact. It emphasizes the interdependence, temporal dynamics, and cultural embeddedness of illness management within partnerships and provides a comprehensive framework for explaining how couples adapt to illness together over time. Building on this theoretical framework, the following hypotheses were derived to examine how different illness types relate to life and relationship satisfaction, and how partnership characteristics may moderate these associations.

### 1.3 Hypotheses

H1: Partnership status moderates the relationship between life satisfaction and an illness. The effects differ depending on the type of illness.

H2: Different illnesses show distinct patterns in their effects on life and relationship satisfaction.

H3: The effect of illness on the relationship is moderated by illness characteristics (severity, duration) and relationship characteristics.

H4: Partnership resources (such as intimacy, religiosity, and conflict resolution patterns) have disease‐specific protective functions.

## 2 Method

### 2.1 Data

This study relies on data from the Family Demographic Panel (FReDA) panel, specifically the data release v.3.0.0, (DOI) https://doi.org/10.4232/1.14080, as published by Bujard et al. (2023). The Family Demographic Panel is a nationally representative study in Germany that surveys individuals aged 18 to 49. The panel addresses a wide variety of issues concerning the lives of families and couples. For a comprehensive overview of the FReDA panel, please refer to Schneider et al. (2021).

This study utilizes data from survey waves W1A and W1B, as well as the additional partner survey (subwave W1A). The data were collected between April and December 2021. Thus, the subwaves were conducted within a maximum interval of about 6 months, with most variables assessed within one to 3 months of each other, allowing them to be treated as approximately cross‐sectional for the purposes of this analysis.

Socio‐demographic variables, partnership variables, and psychosocial variables were obtained from W1A, whereas health‐related variables were drawn from W1B. The first part of the analysis included only data from the index person who originally participated in the survey (37,815). The second part focused on the dyadic perspective and therefore included only participants with corresponding partner interviews (*N* = 14,426).

The selection of variables considered in this analysis was guided by the developmental—contextual model of couples coping with chronic illness proposed by Berg and Upchurch (2007).

1. Health‐related variables included the presence of specific diseases: heart attack, hypertension, cholesterol, stroke, diabetes, lung disease, asthma, cancer, peptic ulcer, Parkinson's disease, cataract, hip fracture, fractures, mental illness, rheumatoid arthritis, osteoarthritis, kidney disease, a category of other diseases and an assessment of general health status. The objective health dimension was approximated through indicators of illness duration, the presence of specific chronic conditions, reported care dependency, and activity limitations. These variables were used to describe the degree of health burden, with higher values suggesting more extensive or prolonged impairments. The subjective health dimension captured respondents' self‐evaluations of their current health and daily life strain, where higher scores indicate a poorer perceived health status. Disease duration captured the self‐reported length of time since diagnosis on a six‐point ordinal scale (0 = *less than 6 months* to 5 = *since childhood*).
2. Socio‐demographic and control variables included age and gender of the individuals, monthly household income, and number of children.
3. Partnership variables included partnership duration, cohabitation, thoughts of separation and partnership satisfaction, sexual activity in the last month, partnership conflict resolution (dysfunctional and functional), division of household tasks (finances, cooking) and satisfaction with the division of labor.
4. Psychosocial variables included life satisfaction/subjective well‐being, fertility intentions, and religiosity.

All key variables were self‐reported using validated scales and collected as part of the panel analysis. A detailed description of the survey methods and the composition of the panel variables can be found in Schneider et al. (2021).

### 2.2 Procedures and Statistical Analyses

The missing values analysis identified systematic missing data in variables collected exclusively for the index person (e.g., illnesses, age of the youngest child), moderate missing data (e.g., income, religiosity), and minimal missing data in the outcome variables (e.g., satisfaction, life satisfaction). Based on this, multiple imputation was carried out with the R package mice (van Buuren and Groothuis‐Oudshoorn 2011). As the disease variables were systematically collected only for the index person, these were excluded from the imputation. The age of the youngest child was also not imputed due to a high proportion of missing values (48.8%), as these presumably occurred systematically in childless couples. The imputation was carried out separately for index persons and partners in order to take the dyadic data structure into account.

Twenty imputed data sets with 50 iterations each were created. Predictive mean matching (PMM) was chosen as the imputation method, as this method better preserves the original distribution of the variables and is robust to violations of the normal distribution assumption (Vink et al. 2014). The plausibility of the imputed values was checked by comparing the distributions of observed and imputed values and by convergence diagnostics (van Buuren 2018). The imputed data sets showed realistic value ranges and distributions for all relevant variables.

For the first part of the analyses, the R packages dplyr (Wickham et al. 2023) and car (Fox and Weisberg 2019), broom (Robinson et al. 2024) were used to compute the Analyses of Variance (ANOVA) and format the output tables. The facet plot was created using ggplot2 (Wickham 2016) and tidyr packages (Wickham et al. 2024). Due to the violation of variance homogeneity, as indicated by the Levene test, a Welch‐ANOVA was applied, as it provides more robust results under heteroscedasticity.

An extension of the Actor‐Partner Interdependence Model (APIM; Kenny et al. 2006) was specified using structural equation modeling (SEM) to investigate the relationships between diseases and partner outcomes. The APIM framework allows for the simultaneous examination of three distinct types of effects in dyadic relationships: (a) crossover effects, which capture how characteristics of one partner affect outcomes of the other partner, (b) spillover effects, which reflect associations between different domains of well‐being within individuals, and (c) partner interdependence, which represents the basic interrelatedness of partner outcomes. While classic APIM studies analyze reciprocal effects of identical variables between partners, our extended model specifies the covariance between relationship and life satisfaction of the non‐ill partner. This methodological modification makes it possible to analyze relationship effects that are not overlaid by their own illness effects. Using this approach, we examined how chronic diseases of one partner (index person) affect both relationship and life satisfaction of their healthy partner.

The structural equation model contains two dependent variables (relationship and life satisfaction), which are predicted by dyadic variables and illness‐related characteristics of the index person. Both direct effects of the illness and interactions with relationship‐related factors (e.g., intimacy, conflict resolution) on both satisfaction measures are modeled. The covariance between the dependent variables was explicitly specified in order to take their relationship into account.

This simultaneous modeling makes it possible to analyze how the relationship dynamics change under illness conditions by examining the effects of dyadic variables on both satisfaction measures of the non‐ill partner without these being confounded by their own illness effects.

The model was specified step by step and contains the following components: Disease‐specific predictors such as the presence of specific diseases (e.g., cancer, mental illness, and kidney disease), regular disease severity and disease duration.

In addition, relationship and psychosocial variables were surveyed. Relationship satisfaction and life satisfaction of the partner were modeled as dependent variables. In addition, interaction effects between disease variables and relationship characteristics were specified in order to examine moderating influences.

The models were estimated using the R package lavaan (Rosseel 2012) using maximum likelihood estimation and robust standard errors (MLR) to account for possible violations of normal distribution assumption. The analyses were based on the data sets previously completed using multiple imputation. In addition to the *χ* ^2^‐test, various fit indices were used to assess the quality of the model: The Comparative Fit Index (CFI), Tucker–Lewis Index (TLI), Root Mean Square Error of Approximation (RMSEA) and Standardized Root Mean Square Residual (SRMR). According to Hu and Bentler (1999), the following criteria were used as cut‐off values for an acceptable fit: CFI/TLI ≥ 0.95, RMSEA ≤ 0.06, SRMR ≤ 0.08.

### 2.3 Participants

The final analysis sample comprised *N* = 3155 dyads consisting of one person with at least one chronic illness (index person) and their partner. The index persons were on average 36.0 years old (SD = 7.9), their partners on average 36.4 years old (SD = 8.8). The couples had been in their relationship for an average of 11.3 years (SD = 7.6), and a high proportion (90.9%) lived in a joint household. The average monthly net household income was 4493 euros (SD = 4775), and the couples had one child on average (*M* = 1.0, SD = 1.1).

The partners' self‐assessed state of health was distributed as follows: 18.0% reported very good health, 52.8% reported good health, 24.4% reported fair health, 4.4% reported poor health and 0.3% reported very poor health. Various chronic illnesses were documented among the index persons: Fractures (36.2%), metabolic diseases (31.0%) and respiratory diseases (20.1%) occurred most frequently, and mental illnesses were present in 19.3% of the index persons. Cardiovascular diseases (3.8%), cancer (3.6%), musculoskeletal diseases (3.5%) and kidney diseases (1.2%) were reported less frequently (for a detailed descriptive overview see Table S1).

## 3 Results

Two complementary analytical strategies were applied. First, an ANOVA examined associations between partnership status (partnered vs. unpartnered) and life satisfaction across different illness groups at the population level. Second, the Actor–Partner Interdependence Model (APIM) focused exclusively on couples and analyzed the partner's perspective, capturing crossover associations between the index person's illness characteristics and the partner's life and relationship satisfaction. Together, these analyses address different but related questions about how partnership context relates to health and subjective well‐being.

### 3.1 ANOVA

**FIGURE 1:**
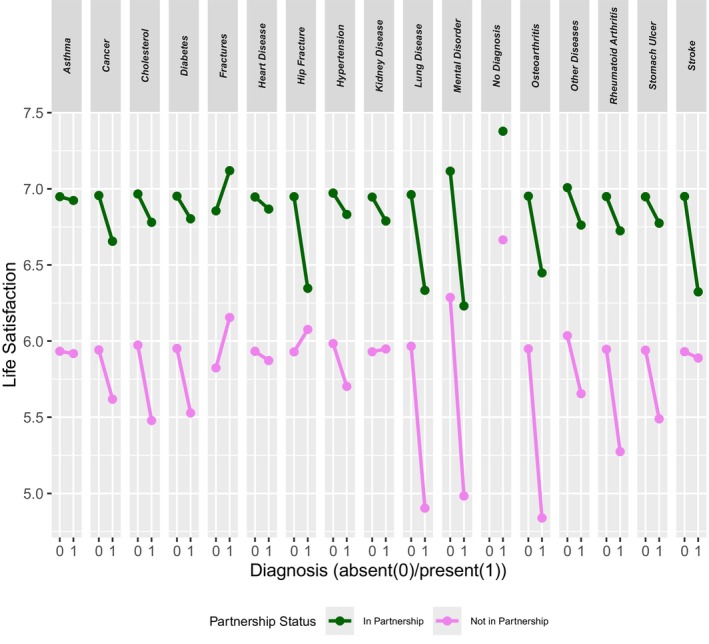
Interaction plot: life satisfaction by disease and partnership status.

Table 1 presents the results of the mean comparisons. The analysis examined the impact of health impairments on life satisfaction, depending on partnership status (see also Figure 1). For this purpose, health impairments were analyzed as an interaction effect with partnership status in a two‐factor ANOVA. Figure 1 illustrates these interaction plots.

**TABLE 1:** Analysis of disease effects on life satisfaction.

Welch's ANOVA						Levene's test				
Disease	df_1_	df_2_	*F*	*η* ^2^		*F*	df_1_	df_2_	*p*	
Heart disease	3	336.26474	134.99	0.000	***	46.33	3	9689	***	
Hypertension	3	1618.01099	138.04	0.001	***	50.09	3	9689	***	
Cholesterol	3	794.40498	138.36	0.000	***	48.41	3	9689	***	
Stroke	3	75.33899	135.02	0.000	***	47.00	3	9689	***	
Diabetes	3	422.52498	136.91	0.000	***	49.52	3	9689	***	
Lung disease	3	268.82662	143.65	0.002	***	47.57	3	9689	***	
Asthma	3	1622.82809	137.10	0.000	***	47.33	3	9689	***	
Cancer	3	316.59654	136.48	0.000	***	47.58	3	9689	***	
Stomach ulcer	3	186.40648	134.55	0.000	***	46.89	3	9689	***	
Hip fracture	3	55.62256	133.38	0.000	***	45.74	3	9689	***	
Fractures	3	2627.35887	155.52	0.003	***	68.76	3	9689	***	
Mental disorder	3	2060.02092	300.71	0.036	***	98.71	3	9689	***	
Rheumatoid arthritis	3	204.36465	137.60	0.001	***	49.33	3	9689	***	
Osteoarthritis	3	142.61369	135.70	0.000	***	47.69	3	9689	***	
Kidney disease	3	142.93776	135.60	0.000	***	47.11	3	9689	***	
Other diseases	3	2218.91581	148.77	0.002	***	59.78	3	9689	***	
No diagnosis	1	3407.36244	284.63	0.015	***	177.65	1	9979	***	

*Note:* Welch's ANOVA was used due to violation of the homogeneity of variance assumption, as confirmed by Levene's test df_1_ = numerator degrees of freedom, df_2_ = denominator degrees of freedom, *η* ^2^ = eta‐squared effect size.

*** *p* < 0.001.

Health impairments related to cataracts and Parkinson's disease were excluded due to small group sizes, and the assumption of normal distribution was confirmed. The Levene's test indicated that the variances across the different disease groups were not equal, violating the assumption of homogeneity. Therefore, a Welch ANOVA was conducted, as it is robust against violations of variance homogeneity. The results show significant interaction effects between partnership status and disease for the following health impairments: heart attack, hypertension, high cholesterol levels, stroke, diabetes, chronic lung disease, asthma, cancer, stomach ulcer, hip fracture, other fractures, anxiety disorder, rheumatoid arthritis, osteoarthritis, chronic kidney disease, other conditions, and no diagnosis (all *p* < 0.001).

The results of the interaction plot (see Figure 1) revealed partially opposing effects of partnership significance depending on specific diseases. Starting from the *no diagnosis* category, a positive influence of partnership on life satisfaction is indicated. This effect is modulated by the presence of various diseases.

For certain conditions (e.g., cancer, high cholesterol levels, heart disease, hypertension), a synchronous pattern emerges, where life satisfaction decreases equally in both groups—those in a partnership and those without. In other conditions, an asynchronous pattern is observed (e.g., mental illnesses, osteoarthritis, hip fractures), where life satisfaction declines more sharply in one group. In some cases, life satisfaction increases with the presence of a disease (e.g., fractures, hip fractures, kidney diseases).

The most significant decline in life satisfaction is found among individuals without a partner who are suffering from chronic lung disease, osteoarthritis, and mental illnesses, as well as among partnered individuals diagnosed with chronic lung disease, mental illnesses, hip fractures, and strokes.

In summary, the impact of diseases is moderated by partnership status, influencing respondents' life satisfaction. These findings highlight the importance of partnership support in coping with health issues and their effects on subjective well‐being, which will be discussed in detail in the following section.

### 3.2 Actor Independence Model

In the next step, the Actor‐Partner Interdependence Model (APIM) was computed (see Table 2). Due to the comprehensive specification of predictors and interaction effects, the APIM was saturated or near‐saturated, resulting in a chi‐square value close to zero with approximately zero degrees of freedom (*χ* ^2^≈0, df≈0, *N* = 6516). This is an expected property of (near‐)saturated models; and resulted in excellent model fit across all disease groups. To account for potential non‐normality, parameters were estimated using maximum likelihood estimation with robust standard errors (MLR). We found significant crossover effects, with disease severity of the index person negatively affecting their partner's relationship satisfaction. Additionally, a substantial within‐partner spillover effect between life satisfaction and relationship satisfaction was observed, indicating a strong interdependence between these two aspects of well‐being.

**TABLE 2:** Actor‐partner interdependence model results by disease group.

Parameter	Mental	Metabolic	Respiratory	Musculoskeletal	Fractures	Digestive	Cancer	Kidney	Cardiovascular
RS‐LS‐Covariance	0.285***	0.283***	0.285***	0.286***	0.288***	0.283***	0.284***	0.287***	0.283***
Disease effect on RS (s1)	−0.107	0.005	−0.238	0.072	0.069	0.170	−0.322*	0.169	−0.174
Disease effect on LS (i1)	−0.032*	0.004	−0.010	0.017	−0.001	−0.004	−0.027*	−0.030*	−0.027
Main effects on partner's RS									
s2 Disease severity (index person)	−0.014	−0.010	−0.007	−0.019	−0.013	−0.011	−0.007	−0.014	−0.016
s3 Disease duration (index person)	−0.051*	−0.063**	−0.059**	−0.059**	−0.063**	−0.059**	−0.062**	−0.060**	−0.057**
s4 Relationship duration	−0.049*	−0.038	−0.055**	−0.052**	−0.049*	−0.060**	−0.057**	−0.050*	−0.055**
s5 Household income	0.005	0.007	0.005	0.008	0.006	0.005	0.007	0.007	0.007
s6 Number of children	−0.079***	−0.068**	−0.074***	−0.074***	−0.063**	−0.070***	−0.068***	−0.077***	−0.079***
Relationship variables on partner's RS									
b1 Partner's religiosity	0.022	0.012	0.007	0.019	0.007	0.020	0.017	0.022	0.021
b2 Partner's cohabitation status	−0.069***	−0.039*	−0.056**	−0.053**	−0.035	−0.052**	−0.055**	−0.047**	−0.052**
b3 Partner's sexual behavior	−0.113***	−0.131***	−0.123***	−0.114***	−0.103***	−0.120***	−0.130***	−0.122***	−0.123***
b4 Partner's separation thoughts	0.388***	0.388***	0.383***	0.390***	0.382***	0.395***	0.384***	0.393***	0.390***
b5 Partner's child desire	0.041*	0.054**	0.036	0.035*	0.040	0.032	0.035*	0.031	0.028
b6 Partner's dysfunctional coping	−0.158***	−0.156***	−0.166***	−0.160***	−0.167***	−0.157***	−0.162***	−0.160***	−0.163***
b7 Partner's satisfaction with division of labor	0.252***	0.243***	0.239***	0.254***	0.249***	0.260***	0.256***	0.252***	0.255***
Control	−0.068***	−0.070***	−0.069***	−0.069***	−0.070***	−0.071***	−0.068***	−0.070***	−0.069***
Interaction effects									
int1 Disease × Relationship Duration	−0.022	−0.028	0.007	−0.013	−0.008	0.032*	0.026	−0.026*	0.011
int2 Disease × Severity	0.010	−0.010	−0.020	0.010	−0.003	−0.006	−0.032*	0.003	0.010
int3 Disease × Partner's Sexual Activity	−0.043	0.051	0.016	−0.132*	−0.070	0.006	0.112**	0.099***	0.030
int4 Disease × Partner's Child Desire	−0.030	−0.053	−0.012	−0.028	−0.022	0.008	−0.006	0.029	0.015
int5 Disease × Partner's Dysfunctional Coping	−0.015	−0.007	0.001	−0.013	0.006	−0.030	0.012	−0.000	0.010
int6 Disease × Partner's Division of Labour Satisfaction	0.010	0.060	0.097	0.025	0.010	−0.191*	−0.053	−0.006	−0.020
int7 Disease × Partner's Religiosity	−0.016	0.022	0.051**	0.029	0.031	0.015	0.027	−0.011	0.023
int8 Disease × Partner's Cohabitation	0.113	−0.099	0.034	0.045	−0.090	−0.018	0.069	−0.365***	0.007
int9 Disease × Partner's Separation Thoughts	0.017	0.044	0.080	−0.000	0.072	0.019	0.197	0.099	0.089
int10 Disease × Number of Children	0.014	−0.012	−0.003	−0.008	−0.023	−0.011	−0.024	−0.001	0.017
int11 Severity × Number of Children	−0.008	−0.000	−0.001	−0.003	−0.004	−0.001	−0.003	−0.002	−0.003
int12 Severity × Partner's Religiosity	0.074***	0.069***	0.066**	0.067**	0.072***	0.066**	0.070***	0.066**	0.065**
int13 Disease duration × Relationship duration	0.039**	0.005	0.031*	0.012	0.020	−0.012	0.012	0.033**	0.005
int14 Disease × Cooking	0.009	0.002	0.008	0.013	0.017	0.013	0.004	0.005	0.006
int15 Disease × Financial Management	0.039**	0.005	0.031*	0.012	0.020	0.012	0.012	0.033**	0.005
int16 Disease × Partner's Functional Coping	−0.016	0.012	0.010	0.023	0.001	0.002	0.030	0.003	0.006
Main effects on partner's LS									
i2 Disease severity (index person)	−0.018	−0.021	−0.020	−0.021	−0.021	−0.021	−0.020	−0.020	−0.020
i3 Disease duration (index person)	−0.048**	−0.056***	0.056***	0.059***	−0.056***	−0.056***	−0.056***	−0.056***	−0.056***
i4 Relationship duration	0.014	0.015	0.015	0.014	0.016	0.016	0.018	0.016	0.016
i5 Household income	0.025*	0.027*	0.027*	0.026*	0.027*	0.027*	0.026*	0.026*	0.026*
i6 Number of children	0.033	0.034	0.034	0.035	0.034	0.034	0.033	0.033	0.033
Relationship effects on partner's LS									
c1 Partner's religiosity	0.058***	0.059***	0.059***	0.059***	0.059***	0.059***	0.058***	0.058***	0.060***
c2 Partner's cohabitation status	−0.055***	−0.055***	−0.055***	0.056***	0.055***	0.056***	0.055***	0.056***	0.055***
c3 Partner's sexual frequency	−0.074***	−0.076***	−0.076***	−0.075***	−0.076***	−0.076***	−0.075***	−0.075***	−0.076***
c4 Partner's separation thoughts	0.198***	0.198***	0.199***	0.199***	0.198***	0.198***	0.199***	0.199***	0.199***
c5 Partner's child desire	−0.020	−0.019	−0.019	−0.019	−0.019	−0.019	−0.020	−0.020	−0.021
c6 Partner's dysfunctional coping	−0.072***	−0.072***	−0.072***	−0.072***	−0.072***	−0.072***	−0.071***	−0.072***	−0.072***
c7 Partner's satisfaction with division of labor	0.179***	0.179***	0.179***	0.179***	0.179***	0.179***	0.179***	0.179***	0.179***
Control	−0.404***	−0.406***	−0.406***	−0.406***	−0.405***	−0.405***	−0.405***	−0.406***	−0.405***
Partner's health status on LS									
CFI	0.997	0.998	0.999	1.000	0.995	0.997	1.000	1.000	0.994
TLI	0.987	0.992	0.996	1.002	0.983	0.987	1.009	1.006	0.979
RMSEA	0.015	0.012	0.009	0.000	0.018	0.015	0.000	0.000	0.020
SRMR	0.002	0.002	0.002	0.002	0.003	0.003	0.001	0.001	0.002
*R* ^2^ RS	0.441	0.439	0.440	0.439	0.438	0.440	0.443	0.440	0.438
*R* ^2^ LS	0.357	0.356	0.356	0.357	0.356	0.356	0.357	0.357	0.357

*Note:* Standardized coefficients are reported. All continuous variables are z‐standardized. Parameter labels: s1–s6: Main effects [s1: Disease presence (index person); s2: Disease severity (index person); s3: Disease duration (index person); s4: Relationship duration; s5: Household income; s6: Number of children]. b1–b7: Relationship variables [b1: Partner's religiosity; b2: Partner's cohabitation status; b3: Partner's sexual behavior; b4: Partner's separation thoughts; b5: Partner's child desire; b6: Partner's dysfunctional coping; b7: Partner's satisfaction with division of labor]. int1–int16: Interaction effects [int1: Disease × Relationship Duration; int2: Disease × Severity; int3: Disease × Partner's Sexual Activity; int4: Disease × Partner's Child Desire; int5: Disease × Partner's Dysfunctional Coping; int6: Disease × Partner's Division of Labor Satisfaction; int7: Disease × Partner's Religiosity; int8: Disease × Partner's Cohabitation; int9: Disease × Partner's Separation Thoughts; int10: Disease × Number of Children; int11: Severity × Number of Children; int12: Severity × Partner's Religiosity; int13: Disease duration × Relationship duration; int14: Disease × Cooking; int15: Disease × Financial management; int16: Disease × Partner's Functional Coping]. i1–i6: Main effects on Partner's Life Satisfaction [i1: Disease presence (index person); i2: Disease severity (index person); i3: Disease duration (index person); i4: Relationship duration; i5: Household income; i6: Number of children]. c1–c7: Relationship effects on Partner's Life Satisfaction [c1: Partner's religiosity, c2: Partner's cohabitation status; c3: Partner's sexual frequency; c4: Partner's separation thoughts; c5: Partner's child desire; c6: Partner's dysfunctional coping; c7: Partner's satisfaction with division of labor]. Control variables: partner's health: effect of partner's own health status on life satisfaction.

* *p* < 0.05.

** *p* < 0.01.

*** *p* < 0.001.

#### 3.2.1 Crossover Effects of Index Person's Disease

The analysis revealed a significant negative effect of disease severity on the partner's relationship satisfaction (s2: *β* = −0.064, SE = 0.013***). This finding suggests that greater disease severity is associated with lower relationship satisfaction. Additionally, a substantial spillover effect between life satisfaction and relationship satisfaction was observed (*β* = 0.705, SE = 0.130***), indicating a strong interdependence between these two aspects of well‐being.

In the analysis of the different diseases, different effect patterns emerged.

For cancer, we observed a direct negative effect on relationship satisfaction (s1: *β* = −0.322*) and the partner's life satisfaction (i1: *β* = −0.027*), along with a significant interaction with disease severity (int2: *β* = −0.032*) and a positive interaction with disease duration (int3: *β* = 0.112**).

For mental illnesses, we found a negative effect on the partner's life satisfaction (i1: *β* = −0.032*), with significant interactions with satisfaction regarding division of household labor (int6: *β* = 0.039**) and a positive interaction between disease severity and religiosity (int12: *β* = 0.074***).

For kidney diseases, we identified a negative effect on the partner's life satisfaction (i1: *β* = −0.030*), along with significant interactions with relationship duration (int1: *β* = −0.026*), disease duration (int3: *β* = 0.099***), cohabitation (int8: *β* = −0.365***), and division of labor (int6: *β* = 0.033**).

#### 3.2.2 Partner Interdependence in Relationship Factors

Several relationship factors showed robust effects across all disease categories. Thoughts of separation (b4: positive association, *β*≈0.38–0.39***), sexual intimacy (b3: negative association, *β*≈−0.11 to −0.13***), satisfaction with division of labor (b7: positive association, *β*≈0.24–0.26***), and dysfunctional conflict resolution (b6: negative association, *β* = −0.16***).

Demographic factors also showed consistent effects. The number of children was negatively associated with relationship satisfaction (s6: *β*≈−0.06 to −0.08**), relationship duration showed small but significant negative effects (s4: *β*≈−0.05*), and disease duration was negatively correlated with satisfaction (s3: *β*≈−0.06**). Additional significant interaction effects emerged in caregiving responsibilities, particularly in mental illnesses (int13: *β* = 0.039**), respiratory diseases (int13: *β* = 0.031*), and kidney diseases (int13: *β* = 0.033**).

#### 3.2.3 Partner Effects

The analysis of effects on the partner's life satisfaction showed consistent patterns across all disease groups. Religiosity (c1: *β*≈0.058–0.060***), cohabitation (c2: *β*≈−0.055***), sexual behavior (c3: *β*≈−0.075***), thoughts of separation (c4: *β*≈0.198–0.199***), dysfunctional conflict resolution (c6: *β*≈−0.072***), and satisfaction with division of labour (c7: *β*≈0.179***).

The main effects on the partner's life satisfaction were particularly evident in disease duration (i3: *β*≈−0.056***) and household income (i5: *β*≈0.025–0.027*), while the effects of disease severity (i2), relationship duration (i4), and number of children (i6) were not significant. A particularly strong and consistent effect was observed for the partner's health status on their own life satisfaction (*β*≈−0.405***). This effect was stable across all disease groups.

The model explained a substantial proportion of variance in both target variables. Forty‐four percent of the variance in relationship satisfaction (*R* ^2^ = 0.44) and 36% of the variance in the partner's life satisfaction (*R* ^2^ = 0.36).

### 3.3 General Summary

The findings provide associative support for most of the proposed hypotheses. H1 was supported by the Welch‐ANOVA suggesting that partnership status is associated with differences in life satisfaction across illness groups. H2–H4 were examined through the APIM analysis, which tested crossover and interaction effects between illness, partner well‐being, and relationship characteristics. Distinct association patterns emerged across diseases: for example, negative links between illness and partner satisfaction in cancer and mental disorders, while weaker or absent associations were found for metabolic disorders. H3 found preliminary support, as several illness characteristics (severity, duration) and relational‐psychosocial factors (e.g., religiosity, sexual activity, cohabitation) showed interaction effects with illness, suggesting that these contexts shape how couples experience health burdens. H4 was partially confirmed. Instead of disease‐specific buffering effects, partnership resources such as conflict resolution, satisfaction with division of labor, and shared religiosity were consistently associated with better well‐being across all illness types. These findings suggest that adaptive mechanisms tend to work more generally within close relationships, rather than being limited to specific health conditions.

## 4 Discussion

These findings demonstrate the complex interplay between illness, partnership characteristics, and well‐being outcomes. While our results confirm the hypothesized relationships, they also reveal nuanced patterns that vary across different types of diseases and partnership resources. Initially, our focus was on disease‐specific effects, assuming that different illnesses would require distinct coping mechanisms within relationships. However, as the analyses progressed, it became evident that many adaptation processes are not confined to specific illnesses but instead follow universal patterns. We explore these findings in detail, evaluating to what extent the original hypotheses were supported and examining their theoretical implications and practical significance for understanding how partnerships adapt to and cope with chronic illness.

### 4.1 The New Normal

Umberson et al. (2010) systematically describe the fundamental mechanisms through which partnerships influence health behavior and outcomes. Their model shows how social relationships shape health through various pathways, including social control, physiological responses, and mental health. In contrast, the absence of such relationships poses a significant health risk, as essential support mechanisms are missing. Our first analysis using Welch‐ANOVA, which explored differences between individuals with and without a partnership, suggests that partnership status is associated with variations in life satisfaction across illness groups. The results indicate that disease‐specific interactions may differentially relate to the type of illness and to partnership status. In some diagnoses, such as stroke or fractures, partnership status seems to relate differently to reported life satisfaction, suggesting that the association between illness and well‐being may vary by relational context.

This suggests that partnership dynamics can be both supportive and burdensome, depending on the type of illness and the couple's coping mechanisms. In the case of acute events that often require intensive caregiving for the affected partner, such as fractures, the immediate adjustment to the changed situation is the primary focus. This phase can significantly impact quality of life and lead to increased stress or conflicts within the partnership. In contrast, long‐term, chronically progressive illnesses require gradual adaptation processes that become deeply integrated into daily life and can shape individual well‐being in the long term (Luttik et al. 2007). Such conditions often have lasting effects on relationship dynamics and can permanently alter role distribution within the partnership (Baanders and Heijmans 2007).

At the same time, the findings suggest that partnership may function as a valuable resource that mitigates disease‐specific symptoms and their impact on life satisfaction, which is also in line with the previously mentioned models by Bodenmann (1995) and Berg and Upchurch (2007). In the present data, this pattern was most noticeable in the context of diabetes, where individuals in partnerships tended to report smaller declines in life satisfaction than those without a partner. This could indicate that partnership has a protective function in stabilizing life satisfaction. A shared approach to health behavior or adjustments in lifestyle within the partnership could play a crucial role in this and have positive effects on well‐being (Nicolucci et al. 2013; Trief et al. 2003). Similar protective associations emerged for mental disorders, where the impact of social support is especially pronounced. Research findings repeatedly confirm that a strong social network is crucial for coping with psychological challenges (Thom et al. 2023). If this support is lacking, it can lead to a significant deterioration in well‐being. Unmarried individuals suffering from mental disorders are particularly vulnerable (Lorant et al. 2003; Umberson and Donnelly 2023).

Another disease‐specific effect becomes apparent in the in‐depth APIM analyses concerning digestive diseases in the context of partnership‐based division of labor. While the partner's satisfaction with the division of labor is generally associated with higher relationship quality (*β*≈0.24–0.26), this association appeared to be weakened in the presence of a digestive disease (*β* = −0.191, *p* < 0.05). This may suggest that the usual mechanisms of partnership‐based division of labor are less related for this group of diseases. The specific symptoms of digestive diseases may lead to particular challenges in daily organization that are more difficult to address through conventional patterns of task distribution. The unpredictability and intensity of the symptoms may require a flexible and situationally adapted redistribution of household responsibilities, calling into question the usual satisfaction with established division‐of‐labor patterns (Calsbeek et al. 2002).

Furthermore, the low visibility of symptoms can make it difficult for partners to fully understand the burden experienced by the affected individual. Moreover, such illnesses are frequently associated with socially taboo or stigmatized symptoms, which may complicate adjustment and empathy within the couple (Chelvanayagam 2014). The lack of visible signs can lead to underestimation of the illness and to reduced acknowledgment of its daily impact (Klettner 2024).

These findings illustrate one aspect of what may become the so‐called new normal in couples coping with invisible illnesses: a redefinition of fairness and mutual support, where conventional indicators of balance, such as division of labor, lose some of their explanatory power for relationship satisfaction.

### 4.2 From Disease‐Specific to Universal Patterns

The aforementioned disease‐specific adaptation processes, ranging from the unique role of partnership in mental illnesses to the specific challenges of digestive diseases, initially suggest that each medical condition requires distinct coping patterns within a partnership. However, the main findings of the APIM reveal a surprising pattern. While previous analyses indicated these disease‐specific differences, a detailed examination of partnership dynamics shows remarkably consistent adaptation mechanisms across different illnesses.

A particularly substantial effect is evident in the spillover from relationship satisfaction to the life satisfaction of the healthy partner (*β*≈0.705). This underscores the central importance of partnership quality for the well‐being of the non‐affected partner. Additionally, the consistently negative control effect of the partner's health status (*β*≈0.405) suggests a strong interdependence between both partners' health conditions. The partner serves as a key source of support and stability but can also be vulnerable, particularly when the relationship is strained by the partner's illness (Umberson et al. 2010). In this context, one's own health is a crucial resource for coping with stress and maintaining well‐being (Reinhardt et al. 2026).

While previous research has often discussed potential gender effects in the relationship between partnership quality and health (Kiecolt‐Glaser and Wilson 2017; Wanic and Kulik 2011), the present study deliberately adopted a dyadic approach that systematically examines partnership effects independently of gender. The substantial variance explanation of 44% in the overall model, as well as the consistent effects across different disease groups, suggests that these are fundamental partnership effects that go beyond gender‐specific perspectives. This interpretation is supported by meta‐analytical findings showing that gender differences in the relationship between partnership quality and health are only marginal (Robles et al. 2014).

The described relationship effects are to be understood as adaptation mechanisms to the situation. This becomes evident, for example, in the interaction of relationship duration with the illness, which showed consistent, mostly negative effects across all diseases. It becomes clear that similar stress patterns emerge with increasing relationship duration (Jowsey 2016). This is also evident in consistent dyadic effects. Thoughts of separation (*β*≈0.38), the influence of sexual intimacy (*β*≈−0.11), and the role of satisfaction with division of labor (*β*≈0.24) follow similar patterns across all disease groups, with the latter highlighting the central importance of practical everyday reorganization.

Further evidence for universal adaptation mechanisms is the consistently negative influence of dysfunctional conflict resolution (*β*≈−0.16, *p* < 0.001). This highlights that dysfunctional communication patterns impair relationship quality similarly across all disease groups, making them a central vulnerability factor in coping with illness (Reinhardt et al. 2026). The fact that functional conflict resolution patterns, unlike dysfunctional ones, do not show significant effects suggests that, in illness coping, avoiding negative communication patterns may be more crucial than the presence of positive strategies. This could be related to the fact that destructive communication is particularly harmful in an already stressful illness situation, whereas constructive communication may be a necessary but not sufficient condition for relationship quality (Kiecolt‐Glaser and Newton 2001).

The interaction between illness severity and religiosity consistently shows positive effects (*β*≈0.065–0.074, *p* < 0.01) across all disease groups, suggesting a universal protective function of religiosity as illness severity increases (George et al. 2002; Rafferty et al. 2015). Regardless of the specific illness, similar adaptation mechanisms appear to take effect. This is further supported by the consistent partner satisfaction covariance (*β*≈0.285), highlighting the strong interconnection between relationship satisfaction and the partner's overall life satisfaction. Paradoxical findings, such as the negative effects of long relationship duration (*β*≈−0.05) or a high number of children (*β*≈−0.07), become the new normal in the context of illness, independent of the specific disease.

The results support key assumptions of resilience research, particularly Walsh's Family Resilience Framework, which emphasizes the importance of shared belief systems, organizational patterns, and communication processes for adapting to chronic illness (Walsh 2002). The consistent effects of religiosity with increasing illness severity, as well as the consistently negative impact of dysfunctional conflict strategies, confirm this theoretical perspective. Particularly relevant is Walsh's emphasis on the interaction between belief systems and practical everyday organization, which is reflected in our data through the consistent importance of satisfaction with division of labor, as well as the effects of intimacy and cohabitation (Walsh 2003).

Furthermore, the findings show that illness coping is strongly shaped by partnership processes. The systematic interconnection between relationship and partner life satisfaction highlights the close link between relationship quality and individual well‐being. This aligns with Johnson's (2019) emotion‐focused perspective, which stresses that secure attachment and emotional accessibility of a partner are central resources for coping with stress, an aspect particularly evident in our data through the significance of intimacy and shared daily life. Bodenmann's (1995) and Bodenmann et al.'s (2016) systemic‐transactional model, which views illness coping as an inherently dyadic process, is also supported by our findings. The universal adaptation mechanisms observed across different illnesses reinforce the assumption that partnership‐based coping is shaped less by specific stressors and more by fundamental relationship processes. These theoretical insights underscore the importance of not only understanding partnership resources theoretically but also actively promoting them in clinical practice.

### 4.3 Clinical Implications

The results have important implications for clinical practice. Rather than focusing primarily on disorder‐specific interventions, there is strong support for a more universal understanding of partnership dynamics under conditions of illness. This supports the call for dyadic treatment approaches that consider the couple as a distinct subsystem (Bodenmann 1995; Pietromonaco et al. 2013). Since the identified adaptation mechanisms largely operate independently of the specific illness, general practitioners can also provide basic partnership‐related recommendations, such as emphasizing the importance of constructive communication or shared everyday organization.

However, a partnership‐based approach is not equally suitable for all couples, as the preference for individual or joint illness coping depends on various factors (Yorgason et al. 2010). Nevertheless, the present findings suggest that such an approach can be beneficial for many couples, particularly when both partners are actively involved in the coping process. A dyadic focus allows for the consideration of both individual needs and the strengths and resources of the relationship.

In the field of cancer, studies already demonstrate the particular effectiveness of couple‐focused counseling approaches compared to individual interventions (Badr et al. 2016, 2015; Badr and Krebs 2013). The present study results show that partner effects are not only relevant in severe illnesses such as cancer, where established psychosocial support services for couples already exist. Even in seemingly more common illnesses, such as digestive disorders or musculoskeletal conditions, there are substantial impacts on relationship quality. This suggests that the existing psycho‐oncological couple support model could serve as a blueprint for other disease areas. Specifically, this means that in cardiovascular diseases, partners should be systematically integrated into rehabilitation programs, particularly regarding the prevention of overprotective behavior. For chronic illnesses like kidney failure, continuous couple counseling services would be beneficial to support the long‐term adjustment of daily routines. In metabolic diseases, lifestyle changes could be explicitly framed as a shared project within the couple.

Even for temporary musculoskeletal impairments, short‐term couple counseling could facilitate role adjustments. Special attention should be given to pain management, which can be seen as a common factor across various illnesses. The way partners respond to and process expressions of pain emotionally could be a key mechanism contributing to relationship strain across different medical conditions (Newton‐John and Williams 2006).

Research shows that a partner's perceptions and beliefs about pain play a crucial role. Uncertainty about the cause of pain can lead to invalidating responses (Burns et al. 2019). Negative partner reactions, such as critical or frustrated remarks, not only reduce relationship satisfaction but are also associated with increased psychological distress and a decline in the patient's functional abilities (Prenevost and Reme 2017). However, these negative effects are not limited to the patient alone; through spillover effects, they can also impact the partner's quality of life. This creates a reciprocal dynamic that can escalate into a self‐reinforcing spiral of emotional stress and interpersonal conflict.

Future studies should therefore focus on how partner uncertainties and negative attribution patterns can be systematically addressed in therapeutic interventions to improve pain‐related communication within relationships (Gagnon et al. 2017). These insights could contribute to the development of cross‐disease support programs that enhance partner communication about pain, reduce uncertainties, and strengthen joint coping mechanisms.

Another significant finding is the role of religion as a resource within relationships. Religion can serve as a stabilizing and meaning‐making factor, contributing to resilience both individually and as a couple. This is particularly relevant as religious beliefs not only provide comfort but also offer a shared framework for interpreting the illness experience. As a result, integrating religious or spiritual elements more explicitly into therapeutic interventions and actively fostering their potential is recommended (Walsh 2008).

Overall, the targeted promotion of internal couple resources strengthens relational bonds and facilitates joint coping with stressful life events. It is beneficial for couples to discuss potential changes in their relationship dynamics early on to identify and resolve misunderstandings and conflicts in a timely manner (Rohrbaugh 2021). In summary, a holistic perspective that incorporates both couple‐centered and spiritual resources represents a promising approach to improving therapeutic practice.

### 4.4 Limitations

While this study provides valuable insights into partnership adaptation processes through its extensive dataset and systematic comparison of different disease groups, several methodological limitations must be considered when interpreting the results.

One important methodological limitation concerns the temporal structure of data collection. Detailed health information was collected exclusively for the index person in the W1B‐subwave, whereas other key relationship variables were recorded for both partners in the W1A subwave a few weeks earlier. A further limitation concerns the inclusion of a small number of time‐sensitive measures, such as sexual activity (past 4 weeks) and family–work conflict (past 3 months). Although the surveys were administered within a short interval, minor temporal mismatches cannot be fully excluded. Moreover, the data used in this study have a cross‐sectional structure, which limits causal interpretation. Particularly in relation to changes within the relationship, a longitudinal data structure would be beneficial. Future research should examine the temporal progression and stability of these variables in more detail, which would also help to better understand causality.

Another methodological limitation is that detailed health information (specific diseases and severity) is only available for the index person. This restricts the ability to investigate partner effects in the context of health limitations. The dyadic nature of the data cannot be fully utilized for these aspects. The disease variables capture only the diagnostic history, not the current health status. This may lead to an overestimation of disease prevalence, while the association with current satisfaction might be underestimated. The analysis of relationships between health and satisfaction must therefore account for this temporal and informational asymmetry, and the observed effects should be interpreted with caution.

At the same time, the population‐based nature of the sample is a particular strength of this study. Illness represents the exception rather than the norm, allowing the findings to reflect subclinical, everyday dynamics in couples rather than clinical disease processes. This design enhances ecological validity, even though the observed associations are correspondingly modest.

## 5 Conclusion

Previous APIM studies primarily focused on reciprocal effects of the same variables between partners. However, our extended approach represents a methodological advancement: by focusing on the healthy partner, we can isolate relational associations without confounding illness‐related influences. This approach enables the investigation of universal partnership mechanisms under conditions of illness, a crucial perspective for understanding the often‐overlooked burden on healthy partners in cases of chronic disease.

A key finding is the surprising universality of partnership adaptation mechanisms, regardless of the specific illness. The consistent relationship effects across all examined diseases suggest that therapeutic approaches should focus less on disease‐specific interventions and more on strengthening fundamental partnership resources. This insight provides valuable impulses for a practice‐oriented and resource‐based development of partnership‐centered treatment strategies.

## Conflicts of Interest

The authors declare no conflicts of interest.

## Supporting information


**Appendix S1:** famp70129‐sup‐0001‐AppendixS1.docx.

## Data Availability

The data that support the findings of this study are available in FReDA at https://www.bib.bund.de/Publikation/2023/Datensatz‐FReDA‐Version=3‐0‐0.html?nn=127496, reference number DOI: https://doi.org/10.4232/1.14080. These data were derived from the following resources available in the public domain: FReDA—Das Familiendemografische Panel, https://search.gesis.org/research_data/ZA7777?doi=10.4232/1.14080.
