# Supplementary material for: Turning Everything Upside Down: The Impact of Illness on Romantic Relationships—A SEM‐Based Actor‐Partner Interdependence Model
Source: Fam Process. 2026 Feb 13;65(1):e70129. doi: 10.1111/famp.70129 (PMC12903840; doi:10.1111/famp.70129)
Supplement: Supplementary file 1 — Appendix S1: famp70129‐sup‐0001‐AppendixS1.docx. [file FAMP-65-0-s001.docx]

| **Table S1**  *Descriptive Statistics by Disease Group* | | | | | | | | | |
| --- | --- | --- | --- | --- | --- | --- | --- | --- | --- |
|  | **Disease Group** | | | | | | | | |
| **Variable** | Mental Disorders | Metabolic Diseases | Respiratory Diseases | Musculo- skeletal | Fractures | Digestive Diseases | Cancer | Kidney Diseases | Cardio- vascular |
| **INDEX PERSON** |  |  |  |  |  |  |  |  |  |
| Age (years) | 35.44 (7.92) | 38.02 (7.59) | 34.74 (7.95) | 39.79 (7.80) | 35.30 (7.60) | 38.09 (7.86) | 40.36 (6.68) | 36.77 (7.73) | 37.75 (7.71) |
| Female | 204 (33.6%) | 470 (57.5%) | 292 (48.3%) | 33 (31.7%) | 664 (58.5%) | 39 (58.2%) | 51 (44.3%) | 21 (53.8%) | 56 (49.1%) |
| Relationship satisfaction (0-10)ᵃ | 8.06 (1.60) | 8.27 (1.48) | 8.37 (1.46) | 8.21 (1.70) | 8.41 (1.39) | 8.42 (1.45) | 8.25 (1.85) | 8.31 (1.44) | 8.41 (1.33) |
| Life satisfaction (0-10)ᵃ | 6.38 (1.81) | 7.17 (1.52) | 7.13 (1.64) | 6.63 (1.84) | 7.36 (1.46) | 6.93 (1.81) | 6.76 (1.89) | 6.82 (1.85) | 6.89 (1.81) |
| Subjective health (1-5)ᵇ | 2.64 (0.86) | 2.38 (0.79) | 2.39 (0.84) | 2.81 (0.80) | 2.19 (0.81) | 2.55 (0.86) | 2.70 (0.88) | 2.67 (0.81) | 2.54 (0.88) |
| Disease severity (0-3)ᶜ | 0.17 (0.47) | 0.08 (0.31) | 0.09 (0.34) | 0.15 (0.43) | 0.06 (0.28) | 0.07 (0.26) | 0.12 (0.33) | 0.16 (0.55) | 0.08 (0.36) |
| Disease duration (0-6)ᵈ | 1.86 (2.03) | 0.73 (1.40) | 1.00 (1.73) | 2.07 (2.05) | 0.74 (1.46) | 1.37 (1.77) | 1.13 (1.72) | 1.15 (1.86) | 1.09 (1.70) |
| Religiosity (0-10)ᵉ | 3.01 (3.03) | 3.30 (3.11) | 2.96 (2.93) | 3.08 (3.01) | 2.97 (2.94) | 3.36 (3.22) | 3.15 (3.20) | 2.44 (2.37) | 2.93 (2.96) |
| Reduced sexual activity (yes)ᶠ | (23.2%) | (22.6%) | (20.7%) | (26.9%) | (17.9%) | (22.4%) | (26.1%) | (23.1%) | (21.1%) |
| Separation thoughts (yes)ᵍ | (23.7%) | (16.4%) | (19.0%) | (20.2%) | (14.9%) | (16.4%) | (13.9%) | (17.9%) | (13.2%) |
| Child desire (1-5)ʰ | 2.65 (1.60) | 2.32 (1.50) | 2.77 (1.58) | 1.96 (1.37) | 2.74 (1.58) | 2.34 (1.56) | 2.07 (1.41) | 2.79 (1.67) | 2.08 (1.42) |
| Dysfunctional conflict resolution (3-15)ⁱ | 5.68 (2.01) | 5.56 (1.97) | 5.51 (1.96) | 5.61 (2.07) | 5.36 (1.82) | 5.93 (2.32) | 5.96 (2.19) | 5.33 (1.72) | 5.50 (1.97) |
| Satisfaction with division of labor (0-10)ᵃ | 7.26 (2.34) | 7.72 (2.11) | 7.65 (2.15) | 7.36 (2.22) | 7.81 (2.06) | 8.22 (1.80) | 7.82 (2.25) | 7.72 (2.28) | 7.53 (2.37) |
| **PARTNER** |  |  |  |  |  |  |  |  |  |
| Age (years) | 36.32 (8.85) | 38.11 (8.38) | 35.31 (8.88) | 40.39 (9.29) | 35.35 (8.28) | 37.36 (7.70) | 40.33 (8.15) | 38.13 (10.19) | 39.32 (9.22) |
| Female | 390 (64.1%) | 350 (42.8%) | 311 (51.5%) | 69 (66.3%) | 480 (42.3%) | 26 (38.8%) | 58 (50.4%) | 17 (43.6%) | 61 (53.5%) |
| Relationship satisfaction (0-10)ᵃ | 8.15 (1.67) | 8.36 (1.56) | 8.37 (1.59) | 8.22 (1.81) | 8.39 (1.56) | 8.28 (1.54) | 8.28 (1.59) | 8.56 (1.55) | 8.21 (1.64) |
| Life satisfaction (0-10)ᵃ | 7.12 (1.89) | 7.43 (1.73) | 7.49 (1.77) | 7.34 (1.77) | 7.53 (1.66) | 7.36 (1.81) | 7.21 (1.83) | 7.21 (1.78) | 7.14 (2.19) |
| Subjective health (1-5)ᵇ | 2.28 (0.82) | 2.23 (0.78) | 2.12 (0.81) | 2.35 (0.73) | 2.10 (0.76) | 2.24 (0.80) | 2.23 (0.87) | 2.05 (0.69) | 2.26 (0.87) |
| Religiosity (0-10)ᵉ | 2.94 (2.99) | 3.31 (3.06) | 3.04 (2.92) | 3.20 (2.98) | 3.04 (2.92) | 2.97 (3.05) | 3.09 (3.18) | 2.82 (2.92) | 3.55 (2.89) |
| Reduced sexual activity (yes)ᶠ | (26.0%) | (24.7%) | (22.5%) | (26.0%) | (22.5%) | (29.9%) | (29.6%) | (28.2%) | (21.1%) |
| Separation thoughts (yes)ᵍ | (18.4%) | (13.8%) | (15.6%) | (17.3%) | (14.7%) | (13.4%) | (10.4%) | (5.1%) | (12.3%) |
| Child desire (1-5)ʰ | 2.61 (1.56) | 2.29 (1.48) | 2.78 (1.59) | 1.99 (1.36) | 2.68 (1.58) | 2.28 (1.62) | 2.20 (1.48) | 2.38 (1.50) | 2.04 (1.37) |
| Dysfunctional conflict resolution (3-15)ⁱ | 5.70 (2.00) | 5.86 (1.95) | 5.67 (1.99) | 5.88 (2.25) | 5.55 (1.86) | 6.03 (1.96) | 5.97 (2.11) | 5.49 (1.67) | 5.61 (1.92) |
| Satisfaction with division of labor (0-10)ᵃ | 7.56 (2.09) | 7.44 (2.24) | 7.58 (2.19) | 7.64 (2.16) | 7.57 (2.12) | 7.70 (2.03) | 7.70 (2.13) | 7.72 (2.31) | 7.72 (2.13) |
| **COUPLE CHARACTERISTICS** |  |  |  |  |  |  |  |  |  |
| Sample size (n) | 608 | 818 | 604 | 104 | 1135 | 67 | 115 | 39 | 114 |
| Relationship duration (years) | 10.18 (7.46) | 12.77 (7.79) | 10.15 (7.27) | 15.08 (8.88) | 10.47 (7.34) | 12.64 (8.52) | 13.97 (8.23) | 11.66 (7.00) | 12.29 (7.96) |
| Cohabitation (yes) | (88.8%) | (94.6%) | (88.6%) | (92.3%) | (89.8%) | (94.0%) | (93.9%) | (100.0%) | (93.0%) |
| Monthly net household income (€) | 3965 (2187) | 4478 (2997) | 4665 (7412) | 5683 (14429) | 4510 (3947) | 4661 (5866) | 4449 (1888) | 4125 (1367) | 4121 (1757) |
| Number of children | 0.83 (1.01) | 1.13 (1.08) | 0.86 (1.04) | 1.13 (1.09) | 0.94 (1.03) | 1.24 (1.17) | 1.01 (1.08) | 0.87 (1.15) | 1.03 (1.18) |

*Note.* Disease Groups: Participants could have multiple chronic conditions. The table shows descriptive statistics for dyads where the index person has at least one condition in the respective disease category. Values are presented as M(*SD*) for continuous variables and n(%) for categorical variables.

Scale Descriptions: ᵃ Higher values indicate greater satisfaction (0 = not at all satisfied, 10 = completely satisfied).

ᵇ 1 = very good, 2 = good, 3 = fair, 4 = poor, 5 = very poor.

ᶜ Composite score: 2 × care dependency + severe limitations (range 0-3.)

ᵈ 0 = no disease, 1 = <6 months, 2 = 6-12 months, 3 = 1-5 years, 4 = 5-10 years, 5 = >10 years, 6 = since childhood.

ᵉ 0 = not at all religious, 10 = very religious.

ᶠ Percentage reporting NO sexual intercourse in the last 4 weeks (1 = yes; 2 = no).

ᵍ Percentage reporting having thought about separation (1 = yes; 2 = no).

ʰ 1 = definitely no, 2 = rather no, 3 = undecided, 4 = rather yes, 5 = definitely yes.

ⁱ Sum of 3 items (withdrawal, stonewalling, criticism), each rated 1-5, higher scores indicate more dysfunctional resolution.
